# The Arduous Path to Diagnosis in a Patient With a Unique Cause of Gastroesophageal Reflux Disease

**DOI:** 10.7759/cureus.21233

**Published:** 2022-01-14

**Authors:** Salman Niaz, Sadaf Zia, Laila Tul Qadar, Mahad M Baig, Saad Khalid

**Affiliations:** 1 Otolaryngology-Head and Neck Surgery, Dow International Medical College, Dow University of Health Sciences, Dow University Hospital, Karachi, PAK; 2 Internal Medicine, Dow University of Health Sciences, Karachi, PAK; 3 Internal Medicine, Dow International Medical College, Dow University of Health Sciences, Karachi, PAK

**Keywords:** hypercalcemia, secondary gerd, brown tumour, adult primary hyperparathyroidism, parathyroid gland adenoma

## Abstract

In the following report, we document a case of gastroesophageal reflux disease (GERD) emerging from a peculiar etiology. A 20-year-old male presented to the out-patient department (OPD) of ear, nose & throat (ENT) of Dow University Hospital in Karachi, Pakistan, by referral from remote hospitals with a complaint of stomach upset. Upon a detailed historical assessment of the patient, the picture of a convoluted path to diagnosis emerged. Considering the patient’s short stature and a bony mass on the mandible, a full body bone scan was ordered, uncovering a brown tumor. An elevated serum parathyroid hormone (PTH) level was detected in the presence of elevated serum calcium and low vitamin D levels. Upon subsequent computed tomography (CT) and magnetic resonance imaging (MRI), a cystic tumor of the pancreas was discovered in addition to a parathyroid adenoma which was promptly operated upon through a right-sided parathyroidectomy. The procedure successfully controlled the serum calcium levels of this patient which are suspected to have produced his gastroesophageal reflux-related symptoms. This case highlights the importance of accessible medical infrastructure and one of the unique causes of GERD.

## Introduction

Gastroesophageal reflux disease (GERD) is a common gastrointestinal motility disorder characterized by heartburn and reflux of gastric contents into the oropharynx. Its prevalence has been estimated to be 18.1%-27.8% in North America and 2.5%-7.8% in East Asia [1]. Gastroesophageal reflux is predominantly attributed to dysfunction of the lower esophageal sphincter (LES). It may also be associated with various other physiological and pathological factors. The most prevalent physiological cause is the brief relaxations of the LES. Other factors include reduced pressure of the LES and impaired gastric emptying [2].

GERD is a common, yet rarely described manifestation of primary hyperparathyroidism (PHPT). Symptoms such as nausea, constipation, and heartburn have been described in PHPT, with heartburn presenting in as many as 30% of patients [3]. The traditional "abdominal groan" associated with PHPT is thought to be caused by the effect of elevated blood calcium levels on intestinal skeletal muscle along with the generation of acid by gastric parietal cells. The exact pathophysiology of gastrointestinal manifestations of PHPT remains poorly elucidated and heavily relies on past research and theories [4]. Diagnosis is crucial, as untreated hyperparathyroidism can result in the development of various complications, including osteoporosis, recurrent fractures, renal osteodystrophy, nephrolithiasis, cardiovascular diseases, and cognitive deficits. The only cure for PHPT is parathyroidectomy, which is indicated in all symptomatic patients [5].

Herein, we present a unique case of primary hyperparathyroidism masquerading in complaints consistent with GERD. This case highlights the diagnostic challenges encountered while dealing with atypical cases of PHPT, especially in low-resource settings where medical and surgical referral is not readily available.

## Case presentation

A 20-year-old male resident of a rural village in Pakistan presented to the out-patient department (OPD) of ear, nose, and throat (ENT) of Dow University Hospital in Karachi, Pakistan, on October 5th, 2021, by referral from remote hospitals with unresolved complaints of stomach pain. According to the patient’s historical account, the patient first noticed his symptoms nine months back in January 2021, at which time he reported them to his primary care provider (PCP). The symptoms included a burning pain in his abdominal area (epigastrium) which was made worse with meals and mainly noticed at night. The patient attempted to resolve the pain by eating lighter meals, but the symptom persisted. A review of systems was unremarkable; furthermore, no known comorbidities were reported to exist within the family. The patient denied any addictions, including tobacco, alcohol, or other recreational drugs, and denied previous surgeries or hospital admissions. 

Upon examination, he appeared to be a young man of short stature and low weight. His vitals were normal. No abnormal lymph nodes were found, and no discoloration of the skin, sclera or mucosal surfaces within the mouth was noted. Additionally, an abdominal examination was equally unremarkable: gut sounds were within acceptable intervals of time within every quadrant, the patient was non-tender upon superficial and deep palpation, an expected absence of guarding was noted, and the liver free-edge was palpated with smooth borders. The lower pole of the right kidney was palpated, while the spleen was unable to be palpated, which are both normal findings in patients of reduced weight. When the PCP’s initial prescriptions of antacids and proton pump inhibitors targeting suspected GERD proved to be minimally effective, the patient was referred to more medically equipped hospitals in the region. 

At such a hospital, history and physical exam, both were repeated, which revealed no significant findings except for the aforementioned short stature and low weight, which documented the patient’s height at 165 centimeters with a weight of 44 kg. A thorough ear, nose, and throat examination was performed, yielding mostly insignificant findings with the exception of a subtle, immobile, non-tender, hard swelling on the patient’s mandible located on the left of the mentum which the patient had not noticed previously. Ear examination showed insignificant findings. Nasal examination was clear and devoid of abnormalities. A throat examination found unremarkable lymph nodes, no abnormal masses, unremarkable palpation of the thyroid upon agglutination, and no abnormal findings. The abnormal bony lesion, in addition to the patient’s short stature, was considered in the potential differential diagnoses, prompting a full body bone scan that uncovered suspicious bone lesions, notably on the ramus of the mandible. Blood analysis of the patient’s serum parathyroid hormone (PTH) level was ordered, resulting in the discovery of drastically elevated PTH levels in the presence of elevated serum calcium and low vitamin D levels (Table 1).

**Table 1:** Patient lab results upon blood analysis PTH: Parathyroid hormone

Test Value	Result	Reference Range
Alkaline Phosphatase (IU/L)	286	44 - 147
Vitamin D3 Level (ng/mL)	17.95	25 - 80
PTH (pg/mL)	986	15 - 65
Serum Calcium (mg/dL)	15.20	8.6 - 10.3
Serum Phosphorus (mmol/L)	1.80	3.0 - 4.5

**Figure 1:**
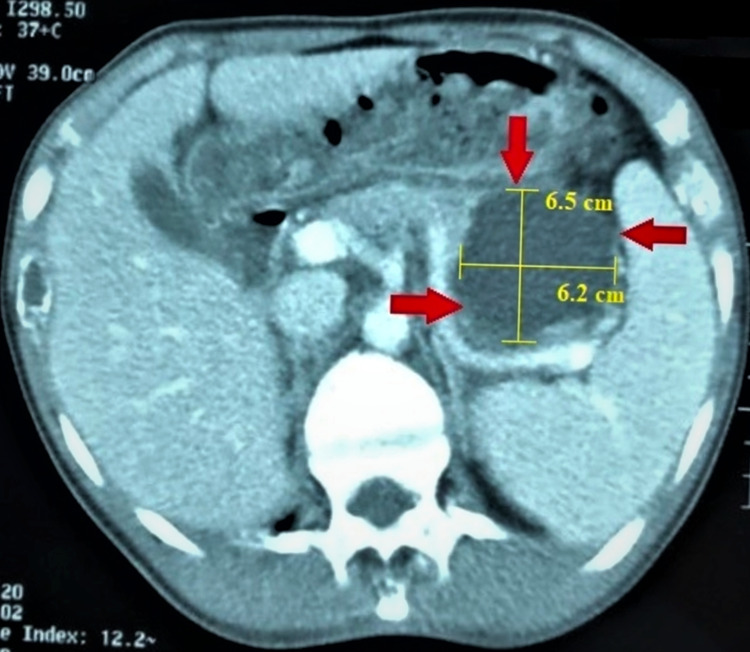
CT scan of abdomen CT: Computed tomography Red arrows highlight the 6.5 cm x 6.2 cm pancreatic pseudocyst evidenced as a loculated cystic lesion identified in the region of the tail of the pancreas extending up to the gastrosplenic ligament

Eventually, the patient was directed to the ENT OPD of Dow University Hospital for radiological imaging studies, since most healthcare centers in the region are not sufficiently equipped for radiological investigation. A gastroenterologist was taken on board. Computed tomography (CT) scan abdomen was suggested. Evidence of a loculated cystic lesion identified in the region of the tail of the pancreas extending up to the gastrosplenic ligament. A well-defined cystic mass measuring 6.5 cm x 6.2 cm, with thin internal enhancing septations was found, which was suggestive of a pancreatic pseudocyst (Figure 1).

**Figure 2:**
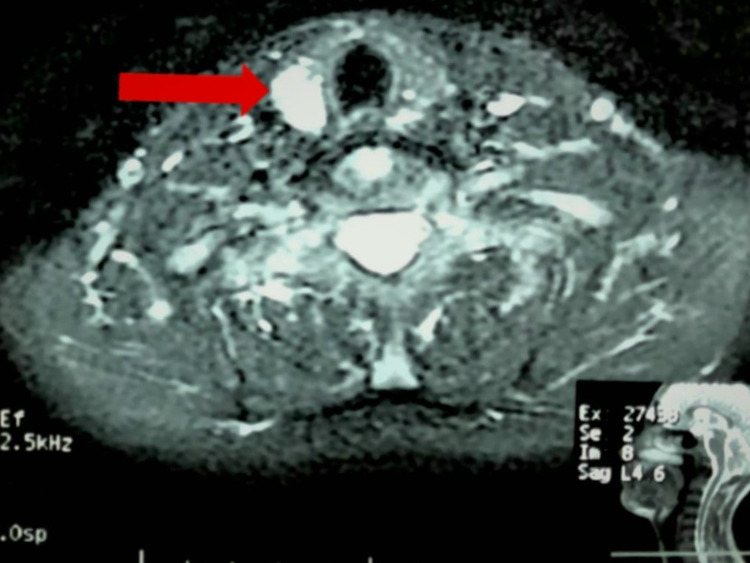
Parathyroid adenoma visualized on MRI contrast of the neck MRI: Magnetic resonance imaging

Additionally, the magnetic resonance imaging (MRI) of the head and neck revealed a parathyroid adenoma (Figure 2), which was promptly operated upon through a successful, uncomplicated, right-sided parathyroidectomy after the patient underwent a sestamibi scan at a local hospital. On the other hand, the pancreatic lesion was not treated since the finding was asymptomatic, and the lesion was not likely to be malignant.

**Figure 3:**
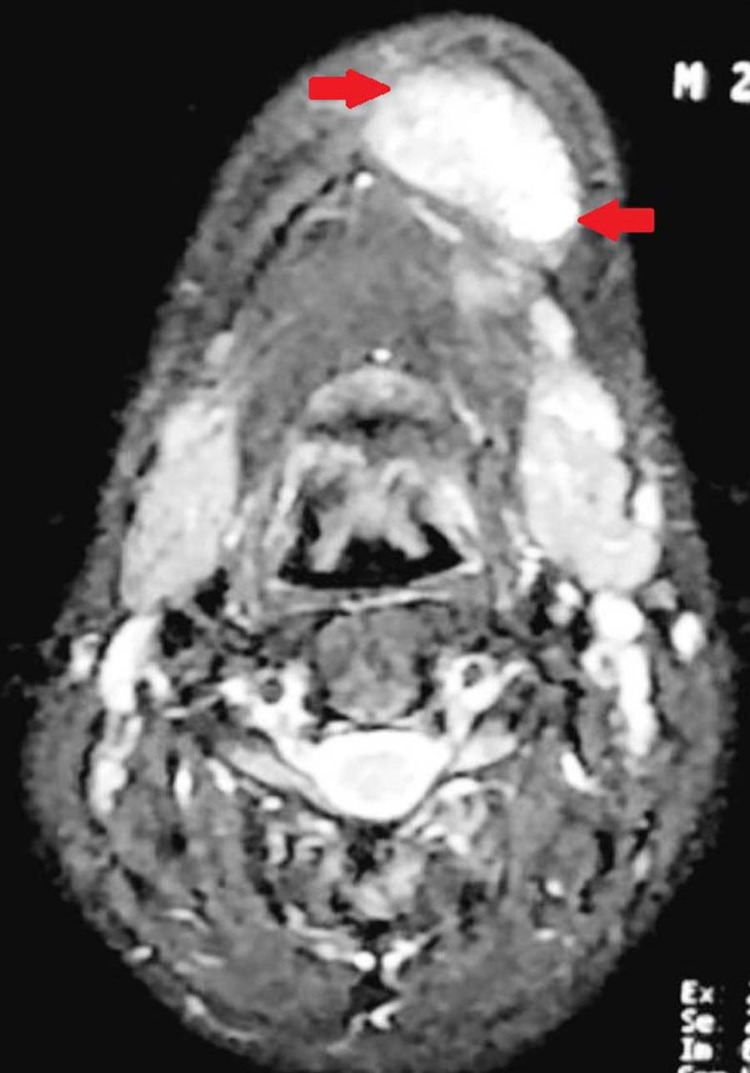
Brown tumor evidenced on MRI localized to the patient's mandible MRI: Magnetic Resonance Imaging

Additionally, the MRI image evidenced the presence of a “brown tumor,” most notably pictured in the mandible of this patient (Figure 3). The parathyroidectomy procedure successfully controlled the serum calcium levels of this patient, which are suspected to have produced his gastroesophageal reflux-related symptoms. The patient is awaited for follow-up. This case highlights the importance of accessible medical infrastructure and one of the unique ways GERD may manifest, leading to a team of ENT specialists treating a patient exhibiting classic gastrointestinal symptoms.

## Discussion

PHPT is a common endocrine abnormality characterized by increased levels of serum PTH and hypercalcemia. PHPT is caused by an increased PTH secretion from one or more of the parathyroid glands. A solitary parathyroid adenoma accounts for 80% of PHPT instances, while four-gland hyperplasia accounts for 10-15%, multiple adenomas for 5%, and parathyroid cancer accounts for around 1%. The prevalence of PHPT is estimated to range from 0.4 to 82 cases per 100,000 [6]. Classical PHPT is a multi-system disorder with skeletal, gastrointestinal, renal, neurological, and psychiatric manifestations. Polyuria, polydipsia, depression, nephrolithiasis, and nephrocalcinosis are common signs and symptoms of this disease. Gastrointestinal manifestations of PHPT include gastric or duodenal ulcer, gastroesophageal reflux disease, constipation, nausea, and decreased appetite, which are all common but infrequently described.

The exact pathophysiology of these symptoms remains unclear [3]. Our patient, although mostly asymptomatic for hyperparathyroidism, presented only with complaints of epigastric burning pain that was treated as GERD initially with PPIs. Atypical cases like these can result in underdiagnosis or delay in diagnosis until the patient presents with fractures or nephrolithiasis later in the course of the disease, adding to the healthcare strain in an already overburdened system. 

Classical PHPT often presents with osteitis fibrosa cystica, a skeletal condition that is characterized by fractures, brown tumors, and bone pain. It remains a frequent mode of presentation in the Middle East, South Africa, and Asia. However, it is uncommon today in the developed part of the world because of routine evaluation of serum calcium [6]. On general examination, our patient’s height appeared lower for his age which was factored into the possible differential diagnoses alongside the boney mass palpated on the mandible. Reduced height and a bony mass on the mandible are indicative of a disorder involving bone formation. These findings prompted a full body bone scan, which revealed multiple brown tumors in the knee, spine, and ramus of the mandible. Further imaging pointed towards the exact source of the patient’s hyperparathyroidism, a parathyroid adenoma.

Another rare association of PHPT with an enigmatic pathophysiological basis is pancreatic pseudocyst, which was diagnosed in our patient. Hypercalcemia is thought to activate acid lysosomal hydrolases, which speeds up the conversion of trypsinogen to trypsin, resulting in pancreatic autodigestion. This incidental pseudocyst could also in part explain his epigastric pain and other symptoms [7].

Parathyroidectomy remains the only standard curative treatment in symptomatic patients with PHPT. Minimally invasive parathyroidectomy is being increasingly adopted throughout the world as it is associated with reduced risk of postoperative pain, shorter hospital stay, and a better aesthetic outcome. However, targeted surgery is contingent on preoperative adenoma localization, which can be accomplished with preoperative ultrasonography or a sestamibi scan. A bilateral neck exploration is sometimes required [8]. However, despite preoperative adenoma localization with a sestamibi scan, our patient underwent right-sided open parathyroidectomy, the reason being the surgeon's preference. The surgical procedure successfully corrected the patient’s calcium levels and ultimately his reflux symptoms. Similar findings were reported in a study by Reiher et al. who demonstrated a significant improvement in GERD symptoms in PHPT patients after parathyroidectomy [3]. 

Although there is room for improvement in the diagnosis and management of patients with PHPT, there is a scarcity of information on how to do so effectively. Studies have shown that most patients do not receive the appropriate diagnostic workup, and only a small percentage of parathyroidectomy candidates receive a surgical referral [9]. Early referral should be considered in all suspected patients presenting with atypical symptoms and increased serum calcium levels to prevent subtle complications of hyperparathyroidism.

## Conclusions

This report sheds light on the arduous path to diagnosis for patients of PHPT. Although most patients of PHPT are asymptomatic, those experiencing atypical symptoms may not reach a proper diagnosis without thorough scrutiny on part of an astute and well-equipped physician. The systemic nature of PHPT symptoms contributes to a large list of differential diagnoses. Narrowing down the list often requires diagnostic procedures which may be unavailable in remote healthcare facilities, especially in developing countries where there is a general lack of expertise accompanying a lack of reliable technology. The patient in our case visited multiple healthcare facilities in order to access diagnostic modalities which ultimately lead to his diagnosis. The publication of case reports documenting initially misleading symptomatology such as that was observed in this patient brings awareness to the potential etiologies which seldom come to mind when evaluating a refractory patient.

## References

[1] El-Serag HB, Sweet S, Winchester CC, Dent J (2014). Update on the epidemiology of gastro-oesophageal reflux disease: a systematic review. Gut.

[2] Clarrett DM, Hachem C (2018). Gastroesophageal reflux disease (GERD). Mo Med.

[3] Reiher AE, Mazeh H, Schaefer S, Gould J, Chen H, Sippel RS (2012). Symptoms of gastroesophageal reflux disease improve after parathyroidectomy. Surgery.

[4] Abboud B, Daher R, Boujaoude J (2011). Digestive manifestations of parathyroid disorders. World J Gastroenterol.

[5] Norman J, Politz D, Lopez J, Boone D, Stojadinovic A (2015). Surgical cure of primary hyperparathyroidism ameliorates gastroesophageal reflux symptoms. World J Surg.

[6] Walker MD, Silverberg SJ (2018). Primary hyperparathyroidism. Nat Rev Endocrinol.

[7] Saif A (2015). Primary hyperparathyroidism presenting with acute pancreatitis and asymptomatic bone involvement. Clin Cases Miner Bone Metab.

[8] Batool S, Shakeel O, Urooj N, Malik AA, Baig M, Ali AA (2019). Management of parathyroid adenoma: an institutional review. J Pak Med Assoc.

[9] Asban A, Dombrowsky A, Mallick R (2019). Failure to diagnose and treat hyperparathyroidism among patients with hypercalcemia: opportunities for intervention at the patient and physician level to increase surgical referral. Oncologist.

